# Supplementary material for: The weekly P25 of the age of the influenza-like illness shows a higher correlation with COVID-19 mortality than rapid tests and could predict the evolution of COVID-19 pandemics in sentinel surveillance, Piura, Perú, 2021
Source: PLoS One. 2024 Mar 7;19(3):e0295309. doi: 10.1371/journal.pone.0295309 (PMC10919873; doi:10.1371/journal.pone.0295309)
Supplement: S3 File — (DOCX) [file pone.0295309.s004.docx]

S4 MINSA Open Data and Knowledge Management in COVID-19, Perú: <https://www.datosabiertos.gob.pe/group/datos-abiertos-de-covid-19>

Through this link, it is possible to access COVID-19 mortality data in Piura, Peru.
