# Supplementary material for: The weekly P25 of the age of the influenza-like illness shows a higher correlation with COVID-19 mortality than rapid tests and could predict the evolution of COVID-19 pandemics in sentinel surveillance, Piura, Perú, 2021
Source: PLoS One. 2024 Mar 7;19(3):e0295309. doi: 10.1371/journal.pone.0295309 (PMC10919873; doi:10.1371/journal.pone.0295309)
Supplement: Attachment — Submitted filename: 1.-Response to Reviewers.pdf [file pone.0295309.s005.pdf]

Response to Reviewers

Dear Editor and reviewers, it is a great pleasure for me to write to you to thank you for your contributions, which have given us the opportunity to improve this manuscript. We have proceeded to follow your instructions and the comments raised have been corrected. In the right column of the following table are the corrections made:

PONE-D-23-01394<br><br>version.	
**Journal requirements:**<br><br>When submitting your revision, we need you to address these additional requirements.	**Authors' corrections**<br><br>Additional requirements that have been addressed
1. Please ensure that your manuscript meets PLOS ONE's style requirements, including those for file naming.	1. The instructions of the manuscript body formatting guidelines and title, author, affiliations formatting guidelines have been strictly followed, achieving compliance with the style requirements of PLOS ONE, including those of file naming.
2. Please provide additional details regarding participant consent. In the ethics statement in the Methods and online submission information, please ensure that you have specified (1) whether consent was informed and (2) what type you obtained (for instance, written or verbal, and if verbal, how it was documented and witnessed). If your study included minors, state whether you obtained consent from parents or guardians. If the need for consent was waived by the ethics committee, please include this information.<br><br>If you are reporting a retrospective study of medical records or archived samples, please ensure that you have discussed whether all data were fully anonymized before you accessed them and/or whether the IRB or ethics committee waived the requirement for informed consent. If patients provided informed written consent to have data from their medical records used in research, please include this information.	2. This study reports retrospective data from the ETI sentinel surveillance system medical records database, which has been collecting data since 2013 anonymously by official order of the health authority; therefore, it was reported in the Material and Methods Section that all the data were completely anonymized before accessing them and the Research Ethics Committee of the Cesar Vallejo University waived the requirement of informed consent. (See lines 172 to 179 of the manuscript)
3. Please update your submission to use the PLOS LaTeX template. The template and	3. The submission has been updated to use the PLOS LaTeX template according to the

more information on our requirements for LaTeX submissions can be found at http://journals.plos.org/plosone/s/latex .	latest LaTeX version of June 2022.
4. Thank you for stating the following financial disclosure:<br><br>“NO”<br><br>At this time, please Addressing the queries:<br><br>a) Please clarify the sources of funding (financial or material support) for your study. List the grants or organizations that supported your study, including funding received from your institution.	4.-Addressing the queries:<br>a) On clarifying the sources of funding (financial or material support) for your study. The organizations that supported our study, including funding received from our institution, were:<br>1. The I - 4 Pachitea Health Center collaborates by providing the facilities to use its infrastructure and access to sentinel surveillance data registered since 2013.<br>2. The Cesar Vallejo University, financed the operating expenses of the research project (support with materials and financing of support staff fees), on the part of the researchers, the cost of their participation is part of the teaching and research activities in the university, and it is covered by the usual monthly salary they receive as teachers.
b) State what role the funders took in the study. If the funders had no role in your study, please state: “The funders had no role in study design, data collection and analysis, decision to publish, or preparation of the manuscript.”<br><br>c) If any authors received a salary from any of your funders, please state which authors and which funders.<br><br>d) If you did not receive any funding for this study, please state: “The authors received no specific funding for this work.”<br><br>Please include your amended statements within your cover letter; we will change the online submission form on your behalf.	b) The funders had no role in study design, data collection and analysis, decision to publish, or preparation of the manuscript.<br><br>c) None of the authors received a salary as a researcher from any of their funders, the activity developed that this study achieved is part of the usual teaching and research activities.<br><br>d) The authors received no specific funding for this work.
5. Thank you for stating the following in the Competing Interests section:	In the Conflict of Interest section, added: "This does not alter our adherence to the PLOS ONE policy on sharing data and materials." (in the

"The opinions expressed in this article belong to the authors and do not necessarily reflect the official policy or position of the Ministry of Health of Peru, Universidad Cesar Vallejo SAC of Peru. Several of the authors are employees of the Peruvian Ministry of Health and/or the Cesar Vallejo SAC University of Peru. This work was prepared as part of his official duties. The authors declare no conflict of interest."<br><br>Please confirm that this does not alter your adherence to all PLOS ONE policies on sharing data and materials, by including the following statement: "This does not alter our adherence to PLOS ONE policies on sharing data and materials." (as detailed online in our guide for authors http://journals.plos.org/plosone/s/competing-interests). If there are restrictions on sharing of data and/or materials, please state these. Please note that we cannot proceed with consideration of your article until this information has been declared.<br><br>Please include your updated Competing Interests statement in your cover letter; we will change the online submission form on your behalf.	same way this has been communicated in the cover letter)
6. Please include your full ethics statement in the 'Methods' section of your manuscript file. In your statement, please include the full name of the IRB or ethics committee who approved or waived your study, as well as whether or not you obtained informed written or verbal consent. If consent was waived for your study, please include this information in your statement as well.	6. The name of the research ethics committee and the waiver of informed consent were added at the end of the Methodology part of the Manuscript (see lines 172 to 179), and our statement.
**Additional Editor Comments:**<br><br>The manuscript has some problems in the description of the methodology and other problems in the language. I request that a comprehensive review be carried out as	Problems in the description of the methodology and other language problems have been addressed. A comprehensive review was carried out as directed by Reviewers 1 and 3. This is presented in the new version of the manuscript.

directed by reviewers 1 and 3.<br><br>**Reviewers' comments:**	
**Reviewer's Responses to Questions**	
**Comments to the Author**	
1. Is the manuscript technically sound, and do the data support the conclusions?<br><br>The manuscript must describe a technically sound piece of scientific research with data that supports the conclusions. Experiments must have been conducted rigorously, with appropriate controls, replication, and sample sizes. The conclusions must be drawn appropriately based on the data presented.<br><br>Reviewer #1: No<br>Reviewer #2: Yes<br>Reviewer #3: Partly	1. The manuscript has been improved to describe technically sound scientific research with data to support the conclusions, data have been added, access to public use databases of COVID-19 mortality (see S1 in the manuscript), and graphs (Fig 1 was added) that help the robustness of the data used. Clarification on appropriate sample sizes. This way, the conclusions were drawn appropriately based on the presented data.
2. Has the statistical analysis been performed appropriately and rigorously?<br><br>Reviewer #1: I Don't Know<br>Reviewer #2: Yes<br>Reviewer #3: I Don't Know	2. The statistical analysis has been carried out adequately according to the measurement scales of the variables, as regards the quantitative variables, normality has been taken into account. This was applied for both descriptive and inferential analysis and is detailed in the methodology.
3. Have the authors made all data underlying the findings in their manuscript fully available?<br><br>The PLOS Data policy requires authors to make all data underlying the findings described in their manuscript fully available without restriction, with rare exception (please refer to the Data Availability Statement in the manuscript PDF file). The data should be provided as part of the manuscript or its supporting information, or deposited to a public repository. For example, in addition to summary statistics, the data points behind means, medians and variance measures should be available. If there are restrictions on publicly sharing data—e.g. participant privacy or use of data	3. The data source is explained in the manuscript in the final part of materials and methods (lines 172 to 180) and a public access link to the mortality and COVID-19 case data has been placed in support S1, it is explained that the ILI database the data is restricted in the access line.

from a third party—those must be specified.<br><br>Reviewer #1: No<br>Reviewer #2: No<br>Reviewer #3: No	
4. Is the manuscript presented in an intelligible fashion and written in standard English?<br><br>PLOS ONE does not copyedit accepted manuscripts, so the language in submitted articles must be clear, correct, and unambiguous. Any typographical or grammatical errors should be corrected at revision, so please note any specific errors here.<br><br>Reviewer #1: No<br>Reviewer #2: Yes<br>Reviewer #3: No	4.- A meticulous correction of the language has been carried out and the language of the article sent has been improved, achieving clarity and in a correct and unequivocal manner. In this way, typographical or grammatical errors in the revision have been specifically corrected.
5. Review Comments to the Author<br><br>Please use the space provided to explain your answers to the questions above. You may also include additional comments for the author, including concerns about dual publication, research ethics, or publication ethics. (Please upload your review as an attachment if it exceeds 20,000 characters)<br><br>Reviewer #1: Please carefully check the entire manuscript for a clear and unambiguous English language. Unfortunately, the presented result, discussion and interpretaion are not clear to me as a general reader. Please look overall for missing and correct words and ensure methodological clarity.<br><br>Reviewer #2: El estudio me pareció interesante, ya que permite observar un comportamiento estacional de la COVID-19, y el debilitamiento de la vigilancia de la Influenza en la región a consecuencia de la pandemia.	5. Response to reviewers:<br><br>To Reviewer #1: That's right, the entire manuscript has been carefully checked for clear and unambiguous English language. Especially in results and discussion have been clarified, methodological clarity is guaranteed.<br><br>To Reviewer #2: Not necessary.

Reviewer #3: In general, the authors make use of very long sentences, difficult to understand. The text must be better redirected, the words sometimes seem unconnected, leaving the text unfluent and confusing.<br><br>Introduction - end of page 11 "and could be equal to or equal to RT-PCR" not understandable.	To Reviewer #3: Short sentences are being used, easy to understand. The text is better redirected, and the words are connected, leaving the text more fluid and understandable. In the Introduction - end of page 11 it was corrected.
Methods – How was your sample defined?	a. The sample studied has been defined as a census sample because it includes all ILI and mortality cases in the study period (see lines 143 to 145).
b. When was the data collection carried out?	b. a census sample of 16,231 registered ILI cases was obtained in May 2022 (see lines 143 to 145).
c. How were possible duplicate cases resolved?	c. The probability of duplicating the data was low because sentinel surveillance was set up to record only incident cases of ILI (see lines 1138 to 142).
d. What was considered "clinical and epidemiological data"?	d. Epidemiological data was considered age, sex, and origin; clinical data was considered such as time of illness, other types of febrile syndrome**,** and pneumonia that they presented were also obtained (see lines 144 to 146.
e. I suggest making a diagram of the different types of information and their quantity (clinical cases, laboratory-confirmed cases, mortality).	e. Figure 1 was added and shows where was obtained the different types of information (see lines 143 to 145)
f. Results - Were the two waves in the country in 2020 and 2021 between exactly the same weeks?	f. Not exactly, the second wave occurred earlier than the first, with approximately two weeks of difference between onset and peaks (see lines 238 to 239).

g. Discussion - Review the description of the acronyms. Absence of period in some sentences and others poorly placed.	g, Discussion- Grammatical errors were corrected in all.
6. PLOS authors have the option to publish the peer review history of their article (what does this mean?). If published, this will include your full peer review and any attached files.<br><br>If you choose "no", your identity will remain anonymous but your review may still be made public.<br><br>Do you want your identity to be public for this peer review? For information about this choice, including consent withdrawal, please see our Privacy Policy.<br><br>Reviewer #1: No<br>Reviewer #2: Yes: Galo Guillermo Farfan Cano<br>Reviewer #3: No	
While revising your submission, please upload your figure files to the Preflight Analysis and Conversion Engine (PACE) digital diagnostic tool, https://pacev2.apexcovantage.com/.	The figures uploaded were edited using PACE.
