# Supplementary material for: The weekly P25 of the age of the influenza-like illness shows a higher correlation with COVID-19 mortality than rapid tests and could predict the evolution of COVID-19 pandemics in sentinel surveillance, Piura, Perú, 2021
Source: PLoS One. 2024 Mar 7;19(3):e0295309. doi: 10.1371/journal.pone.0295309 (PMC10919873; doi:10.1371/journal.pone.0295309)
Supplement: Attachment — Submitted filename: Response to Reviewers.docx [file pone.0295309.s006.docx]

Dear Jie Zhang
Academic Editor
PLOS ONE

We are very grateful for the observations in the manuscript, which have allowed improvements. One of the difficulties that Spanish speakers have is indeed mastery of the English language, which we are trying to overcome in this version of our revised manuscript that is being sent.
The attached table corrects the observations raised, hoping to comply with PLOS ONE’s publication criteria as they currently stand.

Sincerely,

Victor Raúl Ocaña Gutierrez

Corresponding author

| **Observations raised by the editors of PLOS ONE** | **The authors have corrected the observations.** |
| --- | --- |
| PONE-D-23-01394R1  The weekly P25 of the age of the influenza-like illness shows a higher correlation with COVID-19 mortality than rapid tests and could predict the evolution of COVID-19 pandemics in sentinel surveillance, Piura, Peru-2021.  PLOS ONE |  |
| **Journal Requirements:** |  |
| Please review your reference list to ensure that it is complete and correct. If you have cited papers that have been retracted, please include the rationale for doing so in the manuscript text, or remove these references and replace them with relevant current references. Any changes to the reference list should be mentioned in the rebuttal letter that accompanies your revised manuscript. If you need to cite a retracted article, indicate the article’s retracted status in the References list and also include a citation and full reference for the retraction notice. | The reference list was reviewed to ensure it was complete and correct. Retracted references were removed and replaced with relevant current references. Using Zotero software, bibliographic references were updated and replaced with some that were eliminated on the web and were automatically corrected. |
| **Additional Editor Comments:** Reviewer's Responses to Questions |  |
| **Comments to the Author**  1. If the authors have adequately addressed your comments raised in a previous round of review and you feel that this manuscript is now acceptable for publication, you may indicate that here to bypass the “Comments to the Author” section, enter your conflict of interest statement in the “Confidential to Editor” section, and submit your "Accept" recommendation.  Reviewer #1: (No Response) | **Comentarios al autor**  We have accepted the recommendations. |
| 2. Is the manuscript technically sound, and do the data support the conclusions?  The manuscript must describe a technically sound piece of scientific research with data that supports the conclusions. Experiments must have been conducted rigorously, with appropriate controls, replication, and sample sizes. The conclusions must be drawn appropriately based on the data presented.  Reviewer #1: Partly | The manuscript, after careful corrections, manages to describe technically sound scientific research with data that supports the conclusions. It has been shown that the collection and analysis of the data have been carried out rigorously, with adequate sample sizes, using statistical techniques, and using statistic software appropriate to the type of variables to describe, correlate, estimate forecasts, and test hypotheses. This has allowed us to demonstrate more clearly that conclusions were appropriately drawn from the data presented.  From an ethical point of view, the rigor in the conduct of the study has been clarified. |
| 3. Has the statistical analysis been performed appropriately and rigorously?  Reviewer #1: I Don't Know | It has been shown that the collection and analysis of the data have been carried out rigorously, with adequate sample sizes, using statistical techniques, and using statistic software appropriate to the type of variables to describe, correlate, estimate forecasts, and test hypotheses. |
| 4. Have the authors made all data underlying the findings in their manuscript fully available?  The [PLOS Data policy](http://www.plosone.org/static/policies.action#sharing) requires authors to make all data underlying the findings described in their manuscript fully available without restriction, with rare exception (please refer to the Data Availability Statement in the manuscript PDF file). The data should be provided as part of the manuscript or its supporting information, or deposited to a public repository. For example, in addition to summary statistics, the data points behind means, medians and variance measures should be available. If there are restrictions on publicly sharing data—e.g. participant privacy or use of data from a third party—those must be specified.  Reviewer #1: No | The PLOS data policy has been adapted so that all the data underlying the findings described in our manuscript are completely available without restrictions, and the link to access mortality data from the Piura district has been attached to the manuscript. MINSA Open Data and Knowledge Management in COVID-19, Perú: <https://www.minsa.gob.pe/datosabiertos/> Through this link, it is possible to access COVID-19 mortality data in Piura district and COVID-19 cases reported from I-4 Pachitea Health Center, Piura, Peru, and the ILI database has been sent in an Excel.xls file. In addition to the summary statistics, the data tables in the manuscript have placed the data points behind the means, medians, and measures of variance. Indeed, there are restrictions on placing the ILI database publicly, so it has had to be sent in Excel.xls format. |
| 5. ¿El manuscrito se presenta de forma inteligible y está escrito en inglés estándar?  PLOS ONE no edita manuscritos aceptados, por lo que el lenguaje de los artículos enviados debe ser claro, correcto e inequívoco. Cualquier error tipográfico o gramatical debe corregirse en la revisión, así que tenga en cuenta aquí cualquier error específico.  Crítico n.º 1: No | Indeed, one of the great difficulties we have as Spanish speakers is correctly translating what they have written. A maximum effort has been made with the help of English language teachers so that the language manuscript sent is probably clear, correct, and unambiguous. Many typos and grammatical errors have been corrected in the revision, taking into account any specific errors. We are hopeful that the errors will be minimal. |
| 6. Review Comments to the Author  Please use the space provided to explain your answers to the questions above. You may also include additional comments for the author, including concerns about dual publication, research ethics, or publication ethics. (Please upload your review as an attachment if it exceeds 20,000 characters)  Reviewer #1: Although there were some efforts to improve the manuscript, unfortunatelly I still find the sentences difficult to understand. There are several spelling and grammatical errors. I would request to check the entire manuscript, specially for English language and clarity. Also not sure, if serological test (in addition to antigen tests) were used for laboratory diagnosis of SARS-CoV-2. Please confirm | 6. Revisar los comentarios al autor.  A maximum effort has been made with the help of English language teachers so that the language manuscript sent is probably clear, correct, and unambiguous. We check the entire manuscript, any typos and grammatical errors have been corrected in the revision, taking into account any specific errors. We are hopeful that the errors will be minimal. Additionally, we confirmed that no serological tests (other than antigen tests) were used for the laboratory diagnosis of SARS-CoV-2. |
| 7. PLOS authors have the option to publish the peer review history of their article ([what does this mean?](https://journals.plos.org/plosone/s/editorial-and-peer-review-process#loc-peer-review-history)). If published, this will include your full peer review and any attached files.  If you choose “no”, your identity will remain anonymous but your review may still be made public.  **Do you want your identity to be public for this peer review?** For information about this choice, including consent withdrawal, please see our [Privacy Policy](https://www.plos.org/privacy-policy).  Reviewer #1: No | This does not apply to authors |
| RECOMENDACIONES ADICIONALES |  |
| [NOTE: If reviewer comments were submitted as an attachment file, they will be attached to this email and accessible via the submission site. Please log into your account, locate the manuscript record, and check for the action link "View Attachments". If this link does not appear, there are no attachment files.]  While revising your submission, please upload your figure files to the Preflight Analysis and Conversion Engine (PACE) digital diagnostic tool, <https://pacev2.apexcovantage.com/>. PACE helps ensure that figures meet PLOS requirements. To use PACE, you must first register as a user. Registration is free. Then, login and navigate to the UPLOAD tab, where you will find detailed instructions on how to use the tool. If you encounter any issues or have any questions when using PACE, please email PLOS at [figures@plos.org](mailto:figures@plos.org). Please note that Supporting Information files do not need this step. | [NOTA: Si los comentarios del revisor se enviaron como un archivo adjunto, se adjuntarán a este correo electrónico y se podrá acceder a ellos a través del sitio de envío. Inicie sesión en su cuenta, ubique el registro del manuscrito y verifique el enlace de acción "Ver archivos adjuntos". Si este enlace no aparece, no hay archivos adjuntos.]  Mientras revisa su envío, cargue los archivos de su figura en la herramienta de diagnóstico digital Preflight Analysis and Conversion Engine (PACE), <https://pacev2.apexcovantage.com/> . PACE ayuda a garantizar que las cifras cumplan con los requisitos de PLOS. Para utilizar PACE, primero debe registrarse como usuario. La inscripción es gratuita. Luego, inicie sesión y navegue hasta la pestaña CARGAR, donde encontrará instrucciones detalladas sobre cómo usar la herramienta. Si tiene algún problema o pregunta al utilizar PACE, envíe un correo electrónico a PLOS a figure@plos.org. Tenga en cuenta que los archivos de información de respaldo no necesitan este paso. |
|  |  |
